# Pressure Relieving Support Surfaces: a Randomised Evaluation 2 (PRESSURE 2): using photography for blinded central endpoint review

**DOI:** 10.1186/s13063-021-05262-0

**Published:** 2021-04-28

**Authors:** Elizabeth McGinnis, Isabelle L Smith, Howard Collier, Lyn Wilson, Susanne Coleman, Nikki Stubbs, Sarah Brown, Rachael Gilberts, Valerie Henderson, Kay Walker, E. Andrea Nelson, Jane Nixon, on behalf of the PRESSURE 2 Trial Group

**Affiliations:** 1 Clinical Trials Research Unit, University of Leeds, Leeds, UK; 2 Mid Yorks Hospitals NHS Trust, Wakefield, UK; 3 Leeds Community Healthcare NHS Trust, Leeds, UK; 4 Independent Tissue Viability Consultant, London, UK; 5 Pressure Ulcer Research Service User Network, Leeds, UK; 6 Caledonian University, Glasgow, UK

**Keywords:** Pressure ulcer, Wound photography, Blinded outcome assessment, Randomised controlled trial

## Abstract

**Background:**

PRESSURE 2 is a randomised evaluation of the clinical and cost-effectiveness of two types of mattress for the prevention of pressure ulcers (PUs). The primary clinical endpoint was time to development of a category ≥2 PU. The current ‘gold standard’ for PU identification is expert clinical assessment. Due to the mattress appearance, a blinded assessment of the endpoint is not possible. This poses a risk to the internal validity of the study. A possible approach is to use photographs of skin sites, with central blinded review. However, there are practical and scientific concerns including patients’ consent to photographs, burden of data collection, photograph quality, data completeness and comparison of photographs to the current ‘gold standard’. This paper reports the findings of the PRESSURE 2 photographic validation sub-study.

**Method:**

Where consent was obtained, photographs were taken of all category ≥2 PUs on the first presentation to assess over-reporting, and for the assessment of under-reporting, a random sample of 10% patients had an assessment by an independent clinical assessor who also photographed two skin sites. The staff were trained in taking and transferring photographs using standardised procedures and equipment. A card included in the photograph recorded participant details and a ‘greyscale’ for correction of white balance during processing. Three blinded reviewers assessed the photographs and rated how confident they were in their assessment.

**Results:**

The trial recruited 2029 patients; 85% consented to photography, and 532 photographs were received and used in the blinded central review. The level of confidence varied by skin classification with more confidence observed when the skin was assessed as being less severe than a category ≥2 PU. Overall, there was a very good reliability compared to the gold standard expert clinical assessment (87.8%, kappa 0.82).

**Conclusion:**

Study findings have usefully informed the scientific and practical issues of blinded assessment of PU status to reducing the risk of bias in medical device trials. The reliability of central blinded expert photography was found to be ‘very good’ (PABAK). Photographs have been found to be an acceptable method of data validation for participants. Methods to improve the quality of photographs would increase the confidence in the assessments.

**Trial registration:**

ISRCTN Registry ISRCTN01151335. Registered on 19 April 2013

## Background

PRESSURE 2 was a randomised controlled trial (RCT), evaluating the clinical and cost-effectiveness of two types of pressure-relieving mattress frequently used in pressure ulcer prevention practice [1, 2]. Alternating pressure mattresses (APMs) were compared to high-specification foam mattresses (HSFM) with patients at high risk of pressure ulcer (PU) development in acute secondary care settings in the UK. The primary endpoint of the clinical study was the time to development of new category ≥2 PUs [3]; secondary endpoints included time to development of new category ≥1 PUs and new category ≥3 PUs, time to healing of existing category 2 PU and cost-effectiveness. The protocol for the clinical trial and the endpoint validation methods are available elsewhere [4, 5]; the clinical findings are also published [1].

It is recognised that blinding of patients and carers is the ‘Achilles heel’ of most RCTs in wound care [6]. Medical devices such as dressings and pressure-relieving equipment used in wound care and PU prevention differ visually such that it is usually impossible to mask participants. Blinding of outcome assessors can usually be achieved by removing the device prior to the outcome assessment. Unfortunately, while this is possible with a dressing or off-loading device, in most cases, it is not practical or ethical to temporarily move the patient to another mattress for blinded outcome assessment.

The additional challenge is the diagnosis of PUs and in particular pre-clinical markers. Although there are a few studies which identify pre-clinical markers associated with PU development [7–9], the lack of evidence for the pathophysiology of PU and an objective laboratory diagnosis has resulted in the ‘gold standard’ for trial endpoint assessment as clinical assessment by trained research nurses [10, 11].

The diagnosis of a category ≥2 PU can also be subjective. The appearance of a category 2 PU may be similar to other wounds, e.g. those caused by moisture [12, 13]. There is, therefore, a risk of bias if the nurses have an explicit or covert preference for one or other mattress types and misclassify a wound. This could be a threat to the internal validity of the study.

Various alternative approaches to minimise bias and overcome the risk of over- or under-reporting of PU were explored which are reported in the protocol paper [5]. As none of these appeared to be satisfactory, we designed a photography validation sub-study to establish a method of blinded outcome assessment to address both the scientific and practical issues.

## Methods

As full details of the methods are already available [5], a brief summary is now provided.

The main aim of the PRESSURE 2 photography sub-study was to assess the feasibility of using blinded expert central photography review to quantify potential bias in the reporting of the presence of a category ≥2 PU (PRESSURE 2 trial endpoint).

The primary objectives were to assess the following:

1. Over-reporting of PUs category ≥2
2. Under-reporting of PUs category ≥2

The secondary objectives were to assess the following:

1. Rates of consent/potential impact upon trial recruitment
2. Acceptability to patients
3. Compliance with photographs
4. Compliance with the secure transfer of photographs between the research site and the Clinical Trials Research Unit (CTRU)
5. Quality of photographs and confidence of photographic review

Patients at high risk of developing PUs were recruited from acute in-patient facilities.

During recruitment, optional additional consent for photographs was taken. Photographs were taken as follows:

- For all category ≥2 PUs, at the first observation by clinical research nurses (subject to patient consent to photography)
- For a torso and heel pressure area, by an independent clinical assessor from a 10% random sample of patients (subject to patient consent for photography)

As this was exploratory, no formal sample size was calculated. Prior to the start of the study, an estimate of a maximum of 1653 photographs corresponding to 1080 PU photographs and 573 PU-free photographs would be received and reviewed, based on the original trial sample size of 2954 participants, 5.7% pre-existing category 2 PU and 20.5% incidence of category ≥2 PU. As the event rate was lower than anticipated, by the end of the study, an anticipated maximum number of photographs was 918, based on the number of category 2 PUs at baseline reported, new category ≥2 and those taken due to random sample selection.

The choice of camera was made following advice from a professional medical photographer and in consideration of the number needed, budget and ease of use by non-professional photographers. All photographs were taken with the chosen camera model (Canon IXUS 510HS) to ensure consistency of colour and image quality.

Photographs were taken by clinical research nurses and the independent clinical assessors, who had received training in the use of the camera, and they followed the procedures outlined in a work instruction. Photographs were transferred to the CTRU via a secure transfer process and white balance adjusted for variation in lighting conditions using the Adobe® Lightroom software (Adobe Systems Incorporated, San Jose, CA, USA).

A corresponding ‘gold standard’ clinical skin assessment was made by the clinical research nurse and the independent clinical assessor, for comparison with the central expert blind photographic review.

Photograph review sessions were organised; each photograph was simultaneously assessed by 3 of the 4 central blinded expert assessors (SC, LW, NS, EM) to ensure standardisation of image conditions. The photographs (category 2 images from clinical research nurses and 2 skin site images from the independent clinical assessors) were batched together for each central blinded expert session and presented in an ad hoc order. HC oversaw each session and ensured that all photographs were classified by each blinded expert assessor independently and that assessors were blind to the corresponding clinical assessment and the other central blinded expert assessors.

The PU skin assessment is normally a clinical process which includes a holistic assessment and manual examination of the skin. Additionally, the photograph quality can be poor, e.g. dark or blurred. In order to capture uncertainty, the central blinded expert assessors allocated a confidence rating to their skin status decision, using a scale of 0–10 (where 0 = ‘not confident at all’ and 10 = ‘very confident’).

For the assessment against the gold standard clinical assessment, one skin classification was derived from the resulting three central blinded expert assessor classifications. The derived classification was based on the agreement of at least two blinded assessors. All derived central blinded expert assessor classifications were compared against the corresponding clinical assessment.

Analysis of over-reporting is presented as the proportion of agreement between clinical skin assessments by trained clinical research nurses at the scheduled trial assessment visit and the central expert blinded assessor review of category 2 PU photographs.

Analysis of under-reporting is presented as follows:

- The proportion of the agreement between the ‘gold standard’ clinical skin assessment by the independent assessors and the central expert blinded assessor review of photographs taken of the 10% random sample
- The proportion of the agreement between the ‘gold standard’ clinical assessment by the clinical research nurse at the scheduled trial assessment visit and the clinical assessment by the independent assessor at the 10% random sample visits.

Compliance was monitored and adjusted to ensure the 10% proportion of patients were selected.

Where comparisons included all categories of PU, the kappa statistic was calculated using the results from the central blinded expert assessors and the clinical assessments. The kappa statistic is defined as the proportion of agreement after the chance agreement is removed from consideration [14]. In addition, the prevalence and bias-adjusted kappa (PABAK) [15] was also calculated to account for the low prevalence of category 2 PUs and any bias between assessors.

Rates of consent, acceptability of and compliance with photography were reviewed and reported. The quality of photographs and the independent assessors’ confidence were also reported.

## Results

The PRESSURE 2 trial recruited 2029 patients between August 2013 and November 2017. The full clinical results of the trial are already published [1].

At baseline, 145 patients had a total of 177 category 2 PUs. During the trial, 160 patients developed 213 category ≥2 PUs. A maximum of 390 photographs were therefore expected from *n* = 305 patients. Photographs of category ≥2 (at baseline and new events) received were 248 (63.6%): 103 of 180 (57%) expected from the APM arm and 145 of 210 (69%) expected from the HSFM arm. Reasons for the 142 missing photographs were mainly due to the lack of consent, 32.3% were because patients had not provided consent at baseline and 17.5% due to verbal refusal at the time of the photograph request (Table 5).

### Primary objective 1: Over-reporting of PUs category ≥2

#### Central blinded expert photography review versus ‘gold standard’ clinical research nurse clinical assessment

The overall agreement between the central blinded expert photography review assessment and the ‘gold standard’ clinical research nurse clinical assessment of category ≥2 was 83.5% (207/248, 95% CI = (78.9% to 88.1%)). This represents agreement of 88.3% (91/103, 95% CI = (82.1% to 94.5%)) in the APM arm and 80.0% (116/145, 95% CI = (73.5% to 86.5%)) in the HSFM arm (Table 1).

**Table 1:** Summary of blinded central expert review assessments for photographs taken of PU category ≥2

	APM	HSFM	Total
Blinded central expert review assessment			
PU Category ≤1	6 (10.7%)	25 (17.2%)	36 (14.5%)
PU Category ≥2	91 (88.3%)	116 (80.0%)	207 (83.5%)
Unable to determine	1 (1.0%)	4 (2.8%)	5 (2.0%)
Total number of photographs reviewed	103 (100.0%)	145 (100.0%)	248 (100.0%)
Was at least one blinded central expert review assessment PU category ≥ 2?			
Yes	97 (94.2%)	129 (89.0%)	226 (91.1%)
No	6 (5.8%)	16 (11.0%)	22 (8.9%)
Total number of photographs reviewed	103 (100.0%)	145 (100.0%)	248 (100.0%)

### Primary objective 2: Under-reporting of PUs category ≥2

A total of 264 (13.0%) patients were selected for inclusion in the 10% random sample to be assessed by the independent clinical assessor. A maximum of 528 photographs were expected (2 photographs per patient). However, only 167 (63.3%) of these patients had an independent clinical assessment, and of these, 142(85.0%) were reported to have had photographs taken, with a total of 284 (53.8%) photographs returned from 137 (51.9%) patients (Table 2). In terms of compliance with the 10% random sample, the 167 of the patients who had an independent clinical visit equates to 8.2% of the intention-to-treat (ITT) population.

**Table 2:** Summary of independent clinical assessments for a random sample of patients

	APM	HSFM	Overall
Skin verification sub-study visit conducted			
Yes	72 (59.5%)	95 (66.4%)	167 (63.3%)
No	49 (40.5%)	48 (33.6%)	97 (36.7%)
Total	121 (100.0%)	143 (100.0%)	264 (100.0%)
Reason skin verification study visit not done			
Missed by the independent clinical assessor	18 (36.7%)	18 (37.5%)	36 (37.1%)
Participant refused	1 (2.0%)	1 (2.1%)	2 (2.1%)
Participant too unwell	4 (8.2%)	2 (4.2%)	6 (6.2%)
Participant has been transferred to another eligible inpatient facility	1 (2.0%)	0 (0.0%)	1 (1.0%)
Patient has been discharged	9 (18.4%)	15 (31.3%)	24 (24.7%)
Patient has been transferred to an ineligible inpatient facility	1 (2.0%)	1 (2.1%)	2 (2.1%)
Participant has withdrawn from the trial	2 (4.1%)	2 (4.2%)	4 (4.1%)
Participant has died	3 (6.1%)	0 (0.0%)	3 (3.1%)
Did not receive study email	3 (6.1%)	3 (6.3%)	6 (6.2%)
Lack of staff capacity	7 (14.3%)	3 (6.3%)	10 (10.3%)
Reason unknown	0 (0.0%)	2 (4.2%)	2 (2.1%)
Other reasons	0 (0.0%)	1 (2.1%)	1 (1.0%)
Total	49 (100.0%)	48 (100.0%)	97 (100.0%)
If study assessment was conducted, were photographs attempted?			
Yes	63 (87.5%)	79 (83.2%)	142 (85.0%)
No	9 (12.5%)	16 (16.8%)	25 (15.0%)
Total	72 (100.0%)	95 (100.0%)	167 (100.0%
If no photographs were taken, reason:			
Consent for photos to be taken not obtained	9 (100.0%)	9 (56.3%)	18 (72.0%)
Participant no longer wants photos to be taken	0 (0.0%)	3 (18.8%)	3 (12.0%)
Participant does not want photos to be taken at this visit	0 (0.0%)	1 (6.3%)	1 (4.0%)
Not appropriate at this time	0 (0.0%)	2 (12.5%)	2 (8.0%)
Missing	0 (0.0%)	1 (6.3%)	1 (4.0%)
Total	9 (100.0%)	16 (100.0%	25 (100.0%)
Number of photographs received per patient			
0	10 (13.9%)	20 (21.1%)	30 (18.0%)
1	2 (2.8%)	0 (0.0%)	2 (1.2%)
2	57 (79.2%)	67 (70.5%)	124 (74.3%)
3	2 (2.8%)	6 (6.3%)	8 (4.8%)
4	1 (1.4%)	2 (2.1%)	3 (1.8%)
Total number of patients who had verification study assessment	72 (59.5%)	95 (66.4%)	167 (63.3%)

#### Central blinded expert photography review vs independent clinical assessment

There was an agreement between the central blinded expert review photographs and independent clinical assessor in 91.5% of cases (260/284) for all skin site assessments (i.e. healthy, altered or category 1 PU, or category ≥2). This is broken down into 90.5% (114/126) agreement in the APM arm and 92.4% (146/158) agreement in the HSFM arm.

The central blinded expert photograph reviewers identified 15 PUs category ≥2, of which only 6 were also assessed by the independent clinical assessor as a PU category ≥2, 2 were assessed as ‘not applicable’, 7 were assessed as healthy, altered or category 1 PUs (see Table 3). Conversely, there were 10 PUs category ≥2 assessed by the independent clinical assessor; 2 were classified as healthy, altered or category 1 PUs; and 2 were unable to be determined by the central blinded expert photographic review.

**Table 3:** Cross-tabulation of assessment by the independent clinical assessor and blinded expert central photography review

			Blinded expert central photography review				Kappa^k^	PABAK^k^
			Healthy, altered or Cat. 1 PU	Cat. 2, 3, 4 or U	Unable to determine	Overall		
**Overall**	**Independent clinical assessor**	**Healthy, altered or category 1 PU**	254 (89.4%)	7 (2.5%)	3 (1.1%)	264 (93.0%)	0.53	0.93
		**Category 2, 3, 4 or U**	2 (0.7%)	6 (2.1%)	2 (0.7%)	10 (3.5%)		
		**N/A**	4 (1.4%)	2 (0.7%)	1 (0.4%)	7 (2.5%)^l^		
		**Missing**	3 (1.1%)	0 (0.0%)	0 (0.0%)	0 (0.0%)		
		**Overall**	263 (92.6%)	15 (5.3%)	6 (2.1%)	284 (100.0%)		
**APM**	**Independent clinical assessor**	**Healthy, altered or category 1 PU**	110 (87.3%)	3 (2.4%)	1 (0.8%)	114 (90.5%)	0.59	0.92
		**Category 2, 3, 4 or U**	2 (1.6%)	4 (3.2%)	2 (1.6%)	8 (6.4%)		
		**N/A**	1 (0.8%)	1 (0.8%)	0 (0.0%)	2 (1.6%)		
		**Missing**	2 (1.6%)	0 (0.0%)	0 (0.0%)	2 (1.6%)		
		**Overall**	115 (91.3%)	8 (6.4%)	3 (2.4%)	126 (100.0%)		
**HSFM**	**Independent clinical assessor**	**Healthy, altered or category 1 PU**	144 (91.1%)	4 (2.5%)	2 (1.3%)	150 (94.9%)	0.49	0.95
		**Category 2, 3, 4 or U**	0 (0.0%)	2 (1.3%)	0 (0.0%)	2 (1.3%)		
		**N/A**	3 (1.9%)	1 (0.6%)	1 (0.6%)	5 (3.2%)		
		**Missing**	1 (0.6%)	0 (0.0%)	0 (0.0%)	1 (0.6%)		
		**Overall**	**148 (93.1%)**	**7 (4.4%)**	**3 (1.9%)**	**158 (100.0%)**		

*U* unstageable

^k^Kappa and PABAK calculated using only assessments where both the independent assessor and central review assessments were available

^l^IAD/moisture lesion (*N* = 5), dermatological skin condition (*N* = 1) and surgical wound/bruising (*N* = 1)

The kappa statistic of 0.53 is in the region of ‘weak agreement’ [16, 17] however is influenced by a small proportion of PUs category ≥2 reported. The PABAK statistic of 0.93 demonstrates ‘very good agreement’ of photograph assessments compared to clinical assessments.

#### All photographs—central blinded expert photography review vs clinical assessment

The level of agreement between the central blinded expert photograph review and all clinical assessments was 87.8% (467/532). There were 222 PUs category ≥2 assessed on the central blinded expert photographic review, and of these, 213 were also assessed by the clinical assessors (i.e. clinical research nurses and independent clinical assessors) as a PU category ≥2 (2 were assessed as ‘not applicable’ and 7 as healthy, altered or category 1 PUs). Similarly, there were a total of 258 PUs category ≥2 reported by the clinical assessors, of which 38 were classified as healthy, altered or category 1 PUs and 7 were unable to be determined through central blinded expert photography review. The corresponding kappa statistic is 0.82 (‘very good agreement’), and PABAK is equal to 0.82 indicating that photographic assessment has ‘very good agreement’ when compared with expert clinical assessment.

#### Independent clinical assessor versus clinical research nurse skin assessments

Skin assessments by the independent clinical assessor were compared to the clinical research nurse clinical skin assessment which was closest in time. The overall agreement was observed to be 94.6% (157/166) broken down into 91.7% (66/72) agreement for patients in the APM arm and 96.8% (91/94) agreement in the HSFM arm.

There were 12 PUs category ≥2 assessed by the independent assessor, and of these, 5 were reported as healthy, altered or category 1 PUs by the clinical research nurse (see Table 4). When broken down by mattress group, all the category ≥2 PUs reported by the independent clinical assessor but not by the clinical research nurse were in the APM arm. Furthermore, there were 4 skin sites that were assessed as a PU category ≥2 by the clinical research nurse that the independent assessor categorised as healthy, altered or category 1 (1 in the APM arm and 3 in the HSFM arm).

**Table 4:** Cross-tabulation of the overall clinical assessment by independent clinical assessor and clinical research nurse/practitioner (CRN/P)

			Independent assessor			Kappa	PABAK
			Healthy, altered or category 1 PU	Category 2, 3, 4 or unstageable	Overall		
**Overall**	**CRN/P**	**Healthy, altered or category 1 PU**	150 (90.4%)	5 (3.0%)	155 (93.4%)	0.58	0.89
		**Category 2, 3, 4 or unstageable**	4 (2.4%)	7 (4.2%)	11 (6.6%)		
		**Overall**	154 (92.8%)	12 (7.2%)	166^m^ (100.0%)		
**APM**	**CRN/P**	**Healthy, altered or category 1 PU**	61 (84.7%)	5 (6.9%)	66 (91.7%)	0.58	0.83
		**Category 2, 3, 4 or unstageable**	1 (1.4%)	5 (6.9%)	6 (8.3%)		
		**Overall**	62 (86.1%)	10 (13.9%)	72 (100.0%)		
**HSFM**	**CRN/P**	**Healthy, altered or category 1 PU**	89 (94.7%)	0 (0.0%)	89 (94.7%)	0.56	0.94
		**Category 2, 3, 4 or unstageable**	3 (3.2%)	2 (2.1%)	5 (5.3%)		
		**Overall**	92 (97.9%)	2 (2.1%)	94^m^ (100.0%)		

^m^One patient was excluded from the comparison with the clinical research nurse because no baseline or follow-up forms were received

The kappa statistic was observed to be in the region of ‘moderate agreement’; however, this is influenced by the small proportion of PUs category ≥2 observed. The corresponding PABAK statistic of 0.89 overall is in the region of ‘very good agreement’ (see Table 4).

### Secondary objective 1: Rates of consent/potential impact upon trial recruitment

Overall, 1711 (84.3%) patients in the ITT patient population consented to photography. This was comparable in both mattress groups with 860 (84.6%) patients allocated to APM who had consented, and 851 (84.0%) patients allocated to HSFM. There were no patients who reported the photography element as a barrier to trial participation. It was noted that fewer consultees provided additional consent to photographs: 80.8% of those consented by consultees compared to 86.4% of those who provided written or witnessed verbal consent also consented to photographs.

### Secondary objectives 2 and 3: Acceptability to patients and compliance with photographs

There were 170 occasions where photographs of PUs category ≥2 had not been attempted. The reasons for these are summarised in Table 5. The most common reason was consent for photographs had not been obtained (*N* = 56, 32.9%). The reasons were reasonably balanced between the two mattress groups although there was a higher proportion in the HSFM arm where photographs were missed in error (*N* = 14, 14.3%) compared to the APM arm (*N* = 7, 9.7%) (Table 5). In terms of the 10% random sample, of those patients who had an independent clinical assessor visit (*N* = 167), the main reason for non-completion of photographs was because consent had not been obtained (*N* = 18, 72.0%).

**Table 5:** Photography compliance for reported PU category ≥2

	APM	HSFM	Overall
**Reasons for photographs of category ≥ 2 PUs not being taken across all visits (includes re-attempts)**			
Consent for photographs to be taken not obtained	27 (32.1%)	34 (32.4%)	61 (32.3%)
Participants does not want photographs taken at this visit	16 (19.0%)	17 (16.2%)	33 (17.5%)
Not appropriate currently	12 (14.3%)	10 (9.5%)	22 (11.6%)
Missed in error	7 (8.3%)	14 (13.3%)	21 (11.1%)
Photo not taken due to logistical problems (e.g. camera unavailable, not enough time)	7 (8.3%)	8 (7.6%)	15 (7.9%)
Participant no longer wants photographs taken as part of this trial	5 (6.0%)	8 (7.6%)	13 (6.9%)
Pre-photography study set-up	2 (2.4%)	4 (3.8%)	6(.2%)
Reason unknown	2 (2.4%)	4 (3.8%)	6 (3.2%)
Dressing/cast in situ	2 (2.4%)	3 (2.9%)	5 (2.6%)
Camera technical problem	3 (3.6%)	0 (0.0%)	3 (1.6%)
Others	1 (1.2%)	2 (1.9%)	3 (1.6%)
Unable to reposition the patient	0 (0.0%)	1 (1.0%)	1 (0.5%)
**Total**	84 (100.0%)	105 (100.0%)	189 (100.0%)

### Secondary objective 4: Compliance with secure transfer of photographs between the research site and the trial management centre

There were 25 protocol deviations reported relating to the administration of photographs. These were the trial generic email address used for photography transfer (*N* = 11), greyscale card not being in the photograph (*N* = 7), incorrect time of data collection (*N* = 3), photographs received from patients who had not provided initial written consent (*N* = 2), wrong camera used (*N* = 1) and camera stolen (*N* = 1). Where the photographs were transferred using the wrong email address, these were deleted from the senders and receivers email accounts and resent using the secure account. Where photographs were received from patients who provided verbal agreement at the time of the photography but had refused photography during consent to study participation, these were destroyed by the sender and receiver and excluded from the analysis.

### Secondary objective 5: Quality of photographs and confidence in photography assessment

Overall, the reviewers tended to be more confident when they assessed a photograph as healthy, altered or category 1. Reviewer 1 gave a confidence score of at least 6 in 70.5% of cases compared to 55.5% of the photographs they assessed as PU category ≥2. Reviewer 2 had a confidence of at least 6 in 86.5% of photographs they assessed as healthy, altered or category 1 compared to 75.6% of those they assessed as PU category ≥2, and reviewer 3 had a confidence score of at least 6 for 79.5% of the former compared to 68.6% of the photographs they assessed as PU category ≥2. There was a very small number of photographs with no assessment (*n* = 16), and the main reason given for this was the poor quality of the photograph.

## Discussion

A potential imbalance was observed in the over-reporting of PUs between arms indicating that PUs may have been more likely to be over-reported in the HSF arm by the clinical research nurses. However, the confidence intervals for the level of agreement for each group overlap. This needs to be considered alongside the unequal return of photographs of PUs category ≥2, i.e. a lower return rate for the APM arm, and the diagnostic uncertainty associated with central blinded expert photography review.

For the assessment of under-reporting of category≥2 PUs, there was a good agreement between the clinical assessments and the blinded reviewers. However, the results of the independent clinical assessor versus the clinical research nurses when broken down by intervention arm suggest that there may be some under-reporting by both the clinical research nurses and the independent assessor. However, the sample size is too small to determine the level of under-reporting and to distinguish whether there are any differences between the arms. Moreover, there were time intervals between the two clinical assessments. PUs, particularly category 1, are known to develop and resolve within days or even hours [6] which may be reflected in this variation in reporting.

Most patients at the time of recruitment consented to photography. Slightly lower photography consent rates were noted for consultee agreement; this may be due to consultees being protective of the patient if they were uncertain of their wishes.

Patient movements between care environments and changes in capacity and consent were the main contributors to photographs not being taken by the independent clinical assessor. Compliance with the return of photographs was greater than 50% but could have been improved with better processes, e.g. use of improved patient tracking systems. The use of a photography-specific email account may have resulted in some of the protocol violations, and improved methods of secure file transfer are currently being tested.

There were varying levels of confidence from the blinded expert reviewers of the photographs, with more confidence demonstrated in skin sites assessed as healthy, altered or category 1. In only a very small number of photographs could no assessment be made. The quality of the photographs taken was reflected in the central reviewers’ confidence in the assessment. While brief training and detailed work instructions were given to participating sites, the poor quality of some of the images led the team to review the camera settings at sites and during investigator meetings when possible. It was noted that some of the cameras were not set according to the work instruction. Considering the ease of use had informed the camera choice, alternative more user-friendly methods could be considered in the future, e.g. use of mobile phone cameras; however, these would need appropriate methods of data protection and ethical approval. The detailed work instruction for when and how to photograph the skin sites, including camera settings and the use of a greyscale card was designed to standardise and optimise the quality of the photographs. In reality, the work instruction was not always followed, and this was reflected in the quality of the photographs. A process of ensuring the training package had been undertaken by each individual taking the photograph is recommended for the future.

A strength of this study is that agreement between paired assessments from a large number of patients was analysed compared to other studies that have utilised multiple assessments of a small number of photographs [18, 19]. The findings are in line with other inter-rater reliability studies [10, 20, 21] where clinical assessments undertaken by expert assessors are compared; these are summarised in the 2019 International Guidelines for Prevention and Treatment of Pressure Ulcers/Injuries [3]. However, compared to the wider literature, the sampling of all category 2 PUs plus a 10% random sample has resulted in a balance in proportions of normal/altered/category 1 and category 2 skin states, which impacts upon interpretation and kappa results.

A further dimension to consider is that the data was collected and analysed specific to the body site, e.g. sacrum, buttocks and ischial tuberosities, and side of body, e.g. left or right heel. It is known that errors can occur when describing the body sites or transcribing the side of the body [11, 13]. Levels of agreement may be affected by this type of misclassification, and this is being explored in methodological research [22].

It is noted that there was more confidence in the assessment of skin sites which were classified as healthy, altered or category 1 by the blinded expert reviewers. As the endpoint for this study was the development of a category ≥2 PU, these skin categories were grouped together. It is known the consequences for the clinical staff when a patient develops a category ≥2 PU in terms of care quality, performance and investigations [13], and therefore, there is a potential reluctance to take part or recruit patients to a study which uses this endpoint. When considering the analysis design of the future central blinded expert review, it is recommended that relooking at the reliability of category 1 PUs is investigated to assess the impact upon its potential use as a primary endpoint.

The research team set out to assess the potential of over-reporting and under-reporting of PUs of category ≥2, but this cannot really be assessed as the central photographic review is not the gold standard. Rather, the research team were trying to establish if there were differences between the arms in the agreement between central blinded photography review and unblinded clinical assessment that would suggest systematic bias in under- or over-reporting. Overall, ≈15% of clinically assessed PUs of category ≥2 were assessed as normal, altered or category 1 by central blind expert photographic review, and the confidence intervals for the proportion of agreement of PUs of category ≥2 for each group overlapped. One of the concerns in the utility of central blind photographic review was the ability to distinguish between non-blanching erythema and a very early category 2 PU characterised by a small area of epidermal loss within a larger area of erythema [5]. Therefore, differences would be expected between the two assessment methods.

The more concerning finding was that photographic compliance was lower in the APM arm than in the HSFM arm; in future work, return rates require compliance monitoring by trial arm (without compromising trial conduct). It is not clear why the compliance was lower in the APM arm. Further work is required to understand whether this was related to practical difficulties associated with participant movement on the APM or systematic bias.

## Conclusions

The findings of this study have usefully informed the scientific and practical issues of a technique used to identify and reduce the risk of bias in medical device trials in particular when endpoints are changes in skin status and development of wounds. The reliability of central blinded expert photography review was found to be ‘very good’ (PABAK). Photographs have been found to be an acceptable method of data validation for participants to support the ‘gold standard’ clinical assessment. Methods to improve the quality of photographs would increase the confidence in the assessments.

## Data Availability

The datasets used and/or analysed during the current study are available from the corresponding author on reasonable request.

## Abbreviations

APMs: Alternating pressure mattresses
CTRU: Clinical Trials Research Unit
HSFM: High-specification foam mattresses
ITT: Intention-to-treat
PABAK: Prevalence and bias-adjusted kappa
PU: Pressure ulcer
RCT: Randomised controlled trial
